# Successful pregnancy and follow-up after *in vitro* fertilization of a kidney transplant patient with systemic lupus erythematosus, primary biliary cholangitis, and hypothyroidism

**DOI:** 10.5935/1518-0557.20210052

**Published:** 2021

**Authors:** Teresa Gastañaga-Holguera, Marta Calvo, Laura Gómez-Irwin, Isabel Campo Gesto, Virginia González-González, Marta Vidaurreta

**Affiliations:** 1 Assisted Reproduction Department. Hospital Clinico San Carlos, Madrid, Spain; 2 Gastroenterology Department. Hospital Universitario de Cruces, Bizkaia, Spain; 3 Prenatal Diagnosis Department. Hospital Clinico San Carlos, Madrid, Spain

**Keywords:** kidney transplantation, autoimmune/rheumatologic, *in vitro* fertilization, assisted reproductive technology, prepregnancy counseling

## Abstract

This report describes the case of a female kidney transplant patient with systemic lupus erythematosus, primary biliary cholangitis, and postsurgical hypothyroidism due to Grave's disease who had a healthy newborn after *in vitro* fertilization (IVF). Cases of successful pregnancy involving women who underwent IVF after kidney transplantation have been reported. Normal and stable renal function, adequate immunosuppressant therapy, and well-managed blood pressure are requirements to be eligible for IVF and pregnancy. Primary biliary cholangitis without cirrhosis does not seem to worsen during pregnancy and IVF must be individualized in patients with systemic lupus erythematosus. There are no similar case reports involving kidney transplant patients or individuals with autoimmune disorders, so the decision to perform IVF had to be individualized in order to avoid complications for the mother and fetus.

## INTRODUCTION

This report describes the case of a kidney transplant patient with systemic lupus erythematosus (SLE), primary biliary cholangitis (PBC) and postsurgical hypothyroidism submitted to *in vitro* fertilization (IVF) and reviews the literature on the subject.

IVF is an option for kidney transplant patients with infertility. It is important to choose the best moment to perform IVF in these patients, at a time in which the associated autoimmune disorder is inactive.

The most frequent adverse obstetric events in kidney transplant patients are hypertension, proteinuria and preeclampsia in the mother, and prematurity, low birth weight and intrauterine growth restriction in the fetus. Recent data suggest that the risk of graft dysfunction increases after pregnancy, especially when it occurs within two years of transplantation. Mycophenolate mofetil must be switched prior to pregnancy; anti-human leukocyte antigen type II (HLA II) DQ donor specific antibody (DQ-DSA) development may occur due to this change. Pregnancy has been related to graft rejection and significantly shorter graft survival.

The decision to perform IVF was a challenge for the multidisciplinary medical team, since there was no data on the effect of IVF and pregnancy on a patient with the abovementioned comorbidities.

## CASE DESCRIPTION

A nulliparous woman aged 34 years who received a kidney transplant for diffuse lupus nephropathy four years earlier was referred to our center for an IVF assessment. She was amenorrheic and her partner was normozoospermic. The patient was on immunosuppressant therapy with tacrolimus, mycophenolate mofetil and prednisone. She had been diagnosed with PBC with florid duct lesions and had elevated gamma-glutamyl transferase (GGT) and alkaline phosphatase (ALP) levels and positive tests for antinuclear antibodies (ANA) and antimitochondrial antibodies (AMA). She was on intermittent ursodeoxycholic acid (UDCA) therapy. She was diagnosed with hypercholesterolemia and postsurgical hypothyroidism due to Graves´s disease. Her body mass index (BMI) was 17 kg/m^2^.

She smoked twenty cigarettes a day until she was 22 years old and suffered from chronic migraines. She did not drink alcohol. The patient was diagnosed with SLE at the age of 14, with skin and kidney involvement. Seven years later, she suffered progressive and severe deterioration of renal function. As no improvement was observed with immunosuppressant therapy, a first deceased donor transplant was performed three years later with immediate improvement of renal function; however, the patient remained amenorrheic and failed to achieve spontaneous pregnancy. She developed DQ-DSA against HLA II after transplantation when tacrolimus and mycophenolate mofetil were changed to cyclosporine and azathioprine while she attempted to get pregnant.

Her workup revealed the following: follicular stimulating hormone (FSH) 8 IU/L; luteal hormone (LH) 3 IU/L; prolactin 11 IU/L; estradiol 20 pg/mL; thyroid stimulating hormone (TSH) 7.03 µUI/mL. Hormone replacement therapy with levothyroxine was increased to target TSH levels below 2.5 µUI/mL.

The decision to proceed with assisted reproductive technology (ART) treatment involved all the physicians in charge of the patient. The patient and her partner received counseling, underwent risk assessment, and decided to undergo IVF as it was considered the most effective ART therapy for them. She was prescribed a mild stimulation antagonist protocol. Her glomerular filtration rate prior to IVF was 63.5 mL/min, and proteinuria was 0.14 g/24h. Ovarian stimulation was performed with daily 75 international units (IU) of recombinant FSH (Gonal Merck®) as the antral follicle count was adequate and a mild ovarian response was expected. Three ovarian follicles were observed after nine days. Three oocytes were retrieved, and one embryo was obtained after intracytoplasmic sperm injection and transferred into the uterus.

Ongoing pregnancy was achieved. Pregnancy course was favorable and her renal function was stable with normal values of glomerular filtration; proteinuria was not detected in any of the performed control tests. Fetal growth was adequate with the estimated fetal weight (EFW) at 32 weeks residing in the 44th percentile. Fetal growth control at 35 weeks revealed EFW in the 7^th^ percentile, but Doppler ultrasound examination of the umbilical cord portrayed images under the 95^th^ percentile. No other obstetric incidences were observed. Spontaneous amniotic sac rupture occurred at 37 weeks. Labor was induced with vaginal prostaglandins and a vaginal delivery ensued. The baby weighed 2230 g at birth and had an Apgar score of 9/10; arterial umbilical cord pH was 7.38. Breastfeeding was maintained for nine months and was stopped when the patient was restarted on mycophenolate mofetil following her nephrologist´s recommendation, as a high DSA titer was observed. The mother´s HLA antibodies were decreasing after her immunosuppressants were increased. Two years after delivery, the patient had herpes zoster with a dorsal dermatome, which prompted the lowering of the prescribed dose of mycophenolate.

The child is now four years-old and suffers from frequent episodic asthma attacks and is on a treatment with bronchodilators and antileukotrienes. He has no other diseases. Type II HLA antibodies remain positive in the mother, but at a very low titer.

## DISCUSSION

Kidney transplantation has allowed the reestablishment of fertility for many women with end-stage renal disease. ART has been seen as an option for kidney transplant patients with associated infertility. Successful IVF in female kidney transplant patients ^(Yaprak *et al*., 2019; Warzecha *et al*., 2018)^ has been described, but the particularity of the case described in this report is that the patient also had several autoimmune comorbidities, which made the decision to submit the patient to ovarian stimulation for a future pregnancy a dilemma for the medical team. We could not find a similar clinical case in the available literature. Therefore, the evidence of the effects of pregnancy and IVF on patients with each of the comorbidities had to be analyzed separately. It was a shared decision that integrated evidence on pregnancy outcomes and patient preferences.

Kidney transplant patients eligible for IVF must be in good health and present good graft function, adequate immunosuppression and well-managed blood pressure without proteinuria ^(Sarkar *et al*., 2018)^. European guidelines dictate that pregnancy should be attempted at least two years after transplantation, since a shorter time may increase the risk of graft loss ^(Rose *et al*., 2016)^; the guidelines from the American Society of Transplantation recommend that the ideal time to wait until conception is between one and two years. Decreasing immunosuppression during pregnancy and complications such as hypertension, preeclampsia and gestational diabetes that may impair the graft function are more frequent if pregnancy occurs within two years of kidney transplantation. In addition, some studies suggest that postponing pregnancy too many years after transplantation also increases the risk of chronic graft dysfunction and immunological events, which may lead to worse glomerular filtration ^(Kidney Disease: Improving Global Outcomes (KDIGO) Transplant Work Group, 2009)^. However, the decision to offer ART treatment is much more complex and requires consideration of other factors, such as age, general health of the patient, and time since transplantation. Even when circumstances are favorable, a kidney transplant patient is at increased risk of developing hypertension, de novo proteinuria, or worsening of the two if they were preexisting conditions ^(Shah *et al*., 2019)^. Case series describing pregnancies in kidney transplant patients report increases of 35% in the risk of miscarriage in the first trimester and success rates of 90% when pregnancy goes beyond the first trimester. Beyond 24 weeks of pregnancy, the most relevant complications are intrauterine growth restriction and low birth weight (LBW). Our patient's infant had LBW, a finding possibly related to the underlying disease itself ^(Kihara et al., 2018)^. Preterm labor may occur in half of the live births. Vaginal delivery is preferred; cesarean sections should be performed if there are obstetrical indications. Some series reported higher cesarean section rates, possibly due to prematurity or obstetric complications ^(Warzecha *et al*., 2018)^.

The risk of adverse maternal-fetal outcomes appears to be higher in ART pregnancies ^(Cabiddu *et al*., 2018)^, although outcomes of IVF appear to be in the same range of spontaneous pregnancies according to published data. Authors of different studies have pondered about probable bias in the selection of patients for IVF ^(Norrman *et al*., 2015)^. Our patient had good renal function with a normal glomerular filtration rate and no proteinuria. Blood pressure was in the upper limit of the normal range before the start of ART therapy, although the patient did not receive antihypertensive drugs. Her immunosuppressants were switched to cyclosporine, azathioprine, and prednisone, since she could not take mycophenolate mofetil while attempting to get pregnant on account of the drug's potential teratogenicity. These measures are consistent with improving the conditions for conception. Nevertheless, mycophenolate mofetil is more effective at preventing acute graft rejection, and as a result of this change the patient developed de novo DQ-DSA. De novo DSA targets type II HLA of the graft, and its development plays an important role in graft survival. Poor treatment compliance is one of the most important risk factors for the development of de novo DSA. It may occur within the first six months or several years after transplantation and has been associated with graft rejection episodes and significantly shorter graft survival ^(Jung *et al*., 2018)^. It is now well established that DQ antibodies are the most commonly detected de novo post-transplant DSA and have a negative effect on graft survival and function. Detection of DQ-DSA has been associated with the development of chronic tissue injury including chronic antibody mediated rejection ^(Lee *et al*., 2016)^ Three years after the birth of her child, our patient required an increase in the mycophenolate mofetil dose after the detection of a progressive increase in the HLA antibody titer. The HLA-II antibody titer decreased as the dose of immunosuppressants was increased, although as a result she developed herpes zoster infection, a common side effect.

The medium and long-term effects of exposure to immunosuppressants of the fetuses of women submitted to kidney transplantation are still unknown. Populations of T and B cells have been reported to reduce during the first months of life in newborns whose mothers received immunosuppressants, although they normalize within the first year of life ^(Schen *et al*., 2002)^. Clinical long-term impact on the immune system of the child remains unknown. Long-term neurocognitive implication and impact on other organs secondary to use of immunosuppressants seem satisfactory, although they are difficult to distinguish from the effects of the high rates of preterm births observed in mothers submitted to kidney transplantation. Breastfeeding is not contraindicated and mothers should not be discouraged from nursing their infants if the immunosuppressant therapy is based on azathioprine, cyclosporine, tacrolimus, or prednisone. It is well established that infants may be breastfed by mothers taking these medications on account of the lower exposure produced by breast milk compared to intrauterine exposure and the lack of described adverse effects ^(Rentsch, 2020)^.

A consensus statement of the American Society of Transplantation establishes that pregnancy should be avoided if the patient has had active autoimmune diseases within the last six months or has other comorbidities that may potentially impact pregnancy outcomes.

PBC (previously known as primary biliary cirrhosis) suggests an autoimmune pathogenesis in genetically predisposed subjects and environmental triggers ^(Tanaka *et al*., 2018^). There is a strong association with other autoimmune, rheumatic, and systemic disorders such as SLE; associations with Graves-Basedow disease have been described. PBC usually develops slowly, and the natural history of the disease has notably improved since the introduction of treatment with UDCA. Florid duct lesion of the liver is the first histopathologic evidence of primary biliary cholangitis, a finding present in all stages of the disease. Pregnancy does not seem to worsen PBC in patients without cirrhosis, and vaginal delivery is possible ^(Ducarme *et al*., 2014)^. Close monitoring and surveillance are required. In these patients, UDCA therapy may be discontinued, although no side effects or teratogenicity have been described in the offspring of mothers with chronic cholestatic disorders who took UDCA during pregnancy ^(Floreani *et al*., 2015)^.

Previously published results suggest that IVF can be carried out safely in patients with SLE ^(Bellver & Pellicer, 2009)^, although available data come from retrospective series. The most frequent complications described associated to IVF were lupus flare, thrombosis and ovarian hyperstimulation syndrome (OHSS) ^(Orquevaux *et al*., 2015^). ART therapy must be individualized and pregnancy properly planned for when the autoimmune disease is inactive, considering both benefits and possible risks. Our patient did not present lupus flares or thrombotic events.

With the patient in mind, the final medical decision was to prescribe a mild ovarian stimulation protocol in order to obtain a few embryos and avoid OHSS, which would have worsened the patient´s baseline condition ^(Muthuvel *et al*., 2016)^. The objective with this patient was to achieve a single pregnancy. Mild ovarian stimulation to avoid complications has also been described in available literature. We also propose single-embryo transfer in these patients; although multiple pregnancies have been described in published case series, the inherent risk is very high ^(Farr et al., 2014)^ and should be avoided.
